# Postinfection Pig and Ferret Antisera Show Similar Antigenic Profiles for Human Influenza A(H1N1pdm09) Viruses

**DOI:** 10.1111/irv.70261

**Published:** 2026-04-22

**Authors:** Ruth Harvey, Tiphaine Cayol, Basudev Paudyal, Alice Lilley, Christine Carr, Catherine Hatton, Emily Briggs, Rodney S. Daniels, Samuel Richardson, Thomas P. Peacock, Nicola Lewis, Ian Brown, John W. McCauley, Elma Tchilian

**Affiliations:** ^1^ The Worldwide Influenza Centre The Francis Crick Institute London UK; ^2^ The Pirbright Institute Pirbright UK

## Abstract

**Background:**

Monitoring antigenic drift in human influenza A viruses is essential for vaccine strain selection and ensuring protection against circulating strains. Antigenic drift is traditionally assessed using ferret antisera, which provide monospecific responses and human vaccinee sera, which reflect exposure to multiple antigens. In this study, we evaluated the pig as an alternative source of antisera to study antigenic drift compared with immune responses in ferrets and humans. We included seasonal influenza A(H1N1pdm09) human viruses that had shown different antigenic characteristics when using ferret or human antisera.

**Methods:**

Pairs of pigs were inoculated with six human A(H1N1)pdm09 viruses circulating between 2019 and 2023, a period of marked antigenic drift. Pig and ferret antisera raised against these six reference viruses were analysed by haemagglutination inhibition (HI) and virus neutralisation (VN), and homologous and heterologous titre differences were used to assess antigenic reactivity profiles among the viruses between species.

**Results:**

Pigs were successfully infected with all strains, shedding virus and producing antibody responses, confirming their susceptibility to human influenza A viruses. Antigenic reactivity of pig antisera was qualitatively comparable to ferret antisera in both HI and VN assays, although maximum homologous antibody titres were significantly higher in ferrets (on average 16‐fold for HI and from around 12‐ to 210‐fold for VN). The antisera raised against viruses in circulation in 2019 and before, exemplified by A/Guangdong‐Maonan/SWL1536/2019, clade 5a.1, were clearly differentiated by both ferret and pig antisera from those in clade 5a.2 and its derivatives that became predominant.

**Conclusions:**

Ferrets and pigs showed comparable responses and both distinguished clade 5a.1 from clade 5a.2. However, neither model recognised antigenically drifted variants from 2019 to 2022, including subclades 5a.2‐C, 5a.2a‐C.1/C.1.9 and 5a.2a.1‐C.1.1/D, which were distinguishable using human postvaccination antisera.

## 1 Introduction

Human seasonal influenza A viruses continually evolve antigenically necessitating constant monitoring and frequent updating of seasonal influenza vaccine strains. The Global Influenza Surveillance and Response System (GISRS), coordinated through the World Health Organisation (WHO), monitors the genetic and antigenic relatedness of currently circulating viruses, and each year the WHO makes separate recommendations of virus components for inclusion in seasonal influenza vaccines for both the Southern and Northern Hemisphere (SH and NH) [1].

Underpinning the vaccine strain recommendations are phenotypic data characterising the antigenic properties of currently circulating influenza strains relative to previously recommended candidate vaccine viruses (CVVs) and to other reference viruses, including viruses chosen to be representative of emerging genetic groups. Routinely, the ferret is used as a small animal model for antibody responses to human seasonal influenza and reference antisera raised against CVVs and other key reference viruses and used throughout each influenza season in haemagglutination inhibition (HI) and virus neutralisation (VN) assays to assess the antigenic properties of contemporary influenza virus strains. These ferret antisera antigenic analyses are supplemented by human serological response analyses using prevaccination and postvaccination serum panels.

Periodically, disparities have been observed between the relative ability of ferret and human immunological responses to similarly discriminate amino acid substitutions in haemagglutinin (HA), resulting in antigenic evolution, particularly with the A(H1N1pdm09) human influenza viruses. For example, while the ferret antibody responses to 2019 CVVs aligned with the human responses when assessed by HI assays, there were discrepancies with CVVs from 2021 such as A/Sydney/5/2021 (Syd21), where human serology indicated significant antigenic drift commensurate with a need to update the vaccine strain, whereas ferret antisera did not [2, 3].

Pigs are an important host for influenza A viruses and are readily infected by the same H1N1 and H3N2 virus subtypes as humans with periodic transmission in both directions [4]. They also serve as a significant biomedical model for studying human disease [5]. Pigs have a longer life span and are genetically, immunologically, physiologically and anatomically more like humans than small laboratory animals [6, 7]. Additionally, pigs exhibit a comparable distribution of sialic acid receptors in the respiratory tract to humans [8]. Indeed, we have previously shown that pig immune responses following infection with A(H1N1pdm09) influenza virus were similar to those induced in humans [9], thereby providing a model of human influenza infection [10].

Here, we tested whether the pig immunological response was able to discriminate between human A(H1N1pdm09) influenza viruses that might be distinguished by either postinfection ferret antisera or postvaccination human sera.

## 2 Results

### 2.1 Virus Infection of Pigs and Nasal Shedding

**FIGURE 1:**
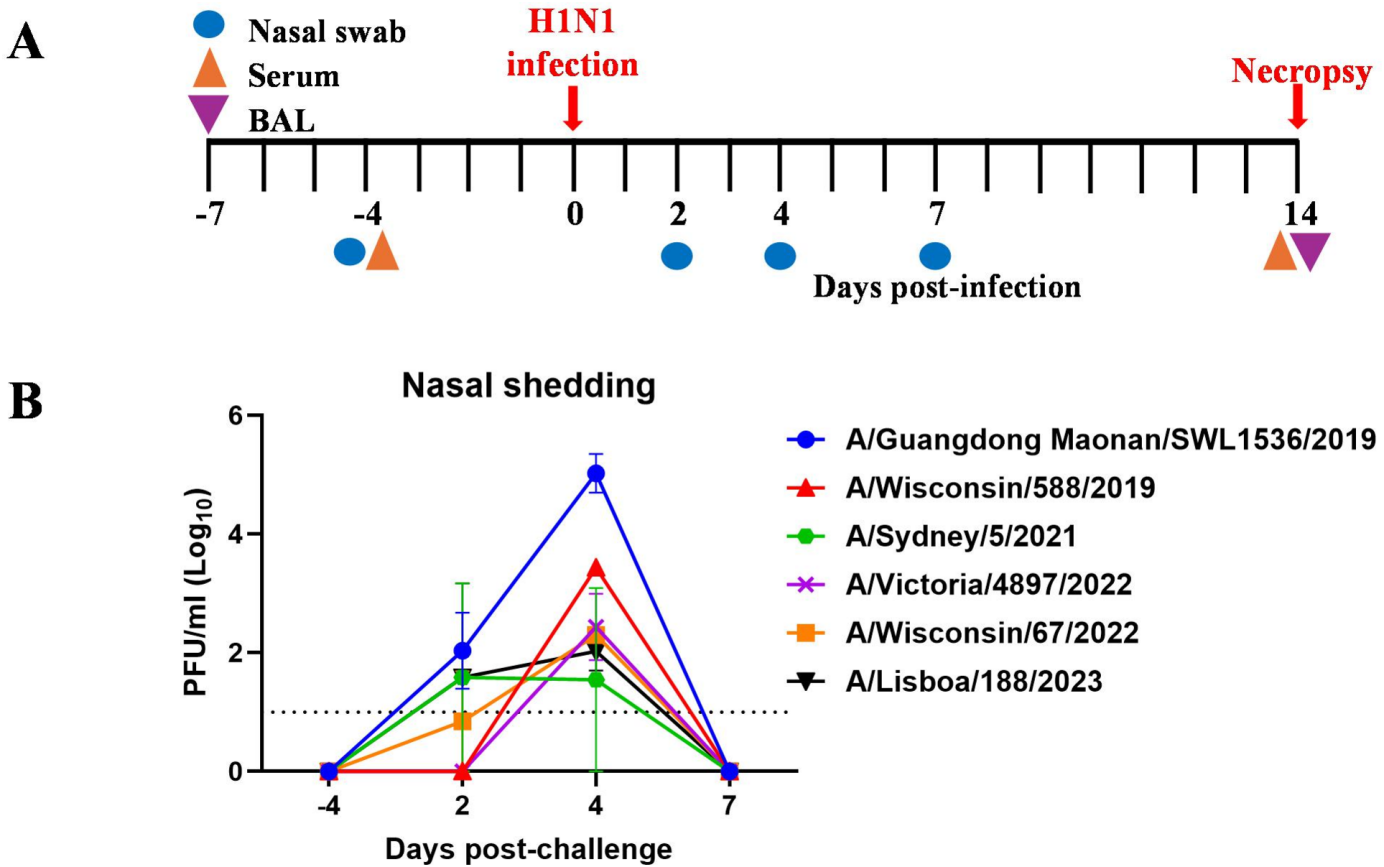
Experimental pig infection and analyses of samples taken. (A) Schematic of the experimental design is shown, two pigs each were intranasally infected with one of the A(H1N1pdm09) viruses shown in Table 1. Serum and nasal swabs were collected at indicated times with necropsy at 14 DPI. (B) Virus loads in nasal swabs at −4, 2, 4 and 7 DPI were determined by plaque assay. Each data point represents the mean of the two pigs inoculated with the same virus and error bars show the standard deviation of that mean. The limit of detection was 1.0 log10 PFU per millilitre, corresponding to one plaque detected in 0.2 mL of sample.

The panel of study viruses selected were representative of viruses in circulation between 2019 and 2023 (Table 1). Over this period, some groups of viruses were differentiated by both postinfection ferret antisera and postvaccination human sera, while others had previously shown detectable differences in response in human serology studies but not when tested against postinfection ferret antisera. Six groups of two pigs each were infected intranasally with one of the study viruses possessing the HA amino acid substitutions indicated (Table 1) and sampled as described in Section 4 and illustrated in Figure 1A. All animals were productively infected as determined by nasal shedding of virus. The infection kinetics showed that mean shedding following infection with all viruses generally peaked at 4 days post‐infection (DPI) (Syd21 infected pigs peaked at 2 DPI) and had been cleared in all pigs by 7 DPI (Figure 1B). Virus whole genome sequencing of the input and 4 DPI nasal swabs (NSs) showed all shed viruses to be unchanged at the consensus level from the infecting viruses, indicating lack of genetic mutations associated with pig infection in a single animal infection cycle (data not shown).

**TABLE 1:** Influenza A(H1N1pdm09) viruses used in this study.

Virus name	Passage history^a^	Clade	Subclade	Vaccination seasons^b^	HA amino acid substitutions compared with previous vaccine strain^c^	
					HA1	HA2
A/Guangdong‐Maonan/SWL1536/2019	C2/M2	5a.1	B	NH 2020/1 (E)	N/A	N/A
A/Wisconsin588/2019	C2/S1/M4	5a.2	C	SH 2021 to NH 2022/3 (C)	K130N, N156K, L161I, A187D, E189Q, V250A^d^	E179D
A/Sydney/5/5/2021	M3/M4	5a.2a	C.1	SH 2023 (E & C)	K54Q, D94N, A186T, *Q189E* , T216A, E224A, R259K, K308R	
A/Lisboa/188/2023	S1/M3	5a.2a	C.1.9	N/A (reference virus)	*N94D* , T120A, K169Q	K58R, I91V, *D179E* , R182K
A/Wisconsin/67/2022	M2	5a.2a.1	C.1.1	NH 2023/4 to NH2025/6 (C)	*N94D* , P137S, K142R, *A216T* , D260E, T277A	E29D, I91V, N124H
A/Victoria/4897/2022	S2/M3	5a.2a.1	D	NH 2023/4 to NH2025/6 (E)	*N94D* , P137S, K142R, D260E, T277A	E29D, I91V, N124H

Abbreviation: N/A = not applicable.

^a^ E = egg, C = cell (specifics unknown), S = SIAT‐1, M = MDCK.

^b^ NH = Northern Hemisphere, SH = Southern Hemisphere, E = egg‐based vaccines, C = cell culture–based and recombinant‐based vaccines.

^c^ Substitutions relate to the previous vaccine virus in the order shown in the table. Those substitutions in italics and underlined indicate amino acid reversions to match a previous vaccine strain. All HA sequences have been submitted to GISAID: GD19 EPI3592700, Wsc19 EPI3571883, Syd21 EPI3363292, Lis23 EPI3363420, Wsc22 EPI3251687, Vic22 EPI3362351.

^d^ A/Wisconsin/588/2019 had A215A/T/I/V polymorphism.

### 2.2 HI Responses of Ferret and Pig Antisera

Antibody titres in HI showed differences in the magnitude of responses between ferret and pig antisera against the panel of study viruses (Table 2). Ferret antisera consistently exhibited high titres of 5120 against the homologous viruses used for infection, indicating a strong antigen‐specific response. In contrast, pig antisera generally demonstrated lower homologous titres, ranging from 160 to 640, depending on the specific virus and individual animal.

**TABLE 2:** Virus neutralising activity of ferret and pig antisera assessed by HI and VN.

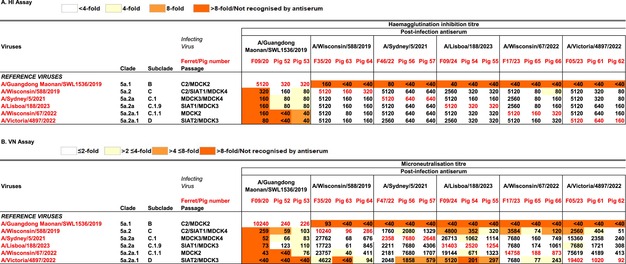

*Note:* Assays, HI(A) and VN (B), were performed as described in Section 4, and the results are ‘heat‐mapped’ in accordance with the respective keys for A and B.

Cross‐reactivity patterns were broadly similar between ferret and pig antisera. The ferret antiserum raised against A/Guangdong‐Maonan/SWL1536/2019 (GM19;subclade 5a.1‐B) showed good recognition of the homologous antigen but very limited recognition of viruses of subclades 5a.2‐C A/Wisconsin/588/2019 (Wsc19), 5A.2a‐C.1 (Syd21), 5A.2a‐C.1.9 A/Lisboa/188/2023 (Lis23), 5a.2a.1‐D A/Victoria/4897/2022 (Vic22) and 5a.2a.1‐C.1.1 A/Wisconsin/67/2022 (Wsc22). Correspondingly, the antisera raised against Wsc19, Syd21, Lis23, Vic22 and Wsc22 all showed high HI titres for the homologous viruses and poor recognition of GM19. However, each of these antisera recognised viruses in subclades 5a.2‐C, 5a.2a‐C.1, 5a.2a‐C.1.9, 5a.2a.1‐D and 5a.2a.1‐C.1.1 at titres within twofold of the homologous titres, most at titres equal to the homologous titre.

While there were some differences in the magnitude of HI antibody titres between individual pigs using the same infecting virus, twofold differences were not considered significant because the antigenic profiles were consistent when comparing serum reactivity between homologous and heterologous viruses for each pair of pigs. One exception was a four fold variation in the homologous titres for Vic22 infected pigs (pigs 61 and 62), but titres to heterologous viruses showed identical reactivity profiles (Table 2A). Like the ferret antiserum raised against GM19, the two pig antisera raised against GM19 recognised the homologous virus well, at HI titres of 320, but recognised the heterologous viruses less well, although pig 53 and pig 54 antisera recognised Wsc19, Syd21 and Lis22 at titres within fourfold lower than the homologous titres. In contrast, all antisera raised against the other five study viruses recognised GM19 poorly, but showed good recognition of the homologous and heterologous viruses, with a single exception for pig 66 (infected with Wsc22) antiserum which recognised Wsc19 at a titre fourfold reduced compared with the homologous titre.

### 2.3 VN of Ferret and Pig Antisera

Ferret antisera raised against GM19 showed a similar clear differentiation in VN assays between the homologous virus and the other five study viruses, as seen by HI assay (Table 2B). Moreover, the antisera raised against the other five study viruses all recognised GM19 poorly. Nevertheless, these antisera showed more variable recognition of the heterologous viruses: those raised against Lis23 (5a.2a‐C.1.9), Wsc22 (5a.2a.1‐C.1.1) and Vic22 (5a.2a.1‐D) all recognised Wsc19 (5a.2‐C) at titres more than fourfold reduced compared with homologous titres, as was the case for Lis23 antiserum with Vic22. Reductions of twofold to fourfold were seen for Wis19 antiserum with Vic22 and Vic22 antiserum with Lis23.

Within pig antisera pairs homologous VN titres ranged from equivalence for GM19 up to fivefold (Wsc22) for four pairs, while for Vic22 (pigs 61 and 62) a 21‐fold difference was seen (Table 2B). Neutralisation titres were consistently higher in ferret antisera for all viruses except Syd21, which had homologous titres of 2358 in ferret and 7680 or 2648 in pigs. Further, the pig antisera showed an even greater variation in recognition patterns in the VN assay than was seen in the HI assay. However, heterologous titres were inconsistent between pig pairs in their profile of reactivity when directly comparing to homologous titres for all infecting viruses.

Again, pig antisera differentiated GM19 (clade 5a.1) from the five viruses in clade 5a.2 and derivatives thereof, antisera raised against them recognising GM19 poorly. Some neutralisation of the heterologous viruses was seen by the antisera raised against GM19, five of the 10 heterologous titres being within fourfold of the homologous titres, one within twofold. Of the 40 heterologous titres associated with antisera raised against the other five study viruses, seven were more than fourfold reduced: five related to Vic22 with antisera raised against Wsc19 (pig 63, with a low homologous titre), Syd21 (both pigs) and Lis23 (both pigs), and two to Wsc19 for antisera raised against Lis23 (pig 54) and Wsc22 (pig 66). Notably, Wsc19 was also recognised poorly by ferret antisera raised against Lis23 and Wsc22.

Overall, antisera raised in both pigs and ferrets against viruses in clades 5a.2, 5a.2a and 5a.2a.1 recognised the 5a.1 virus, GM19, poorly in both HI and VN assays. The antiserum raised against GM19 in ferrets recognised the 5a.2, 5a.2a and 5a.2a.1 viruses poorly in both assays, but the antisera raised in pigs recognised some of the viruses from the heterologous subclades at variable titres, many at titres within fourfold to eightfold of the titres for the homologous antigen. Antisera in ferrets and pigs raised against 5a.2, 5a.2a and 5a.2a.1 viruses recognised viruses in the same clades well, all at titres within fourfold of the homologous titres, all but one within twofold, in HI assays. There was more variation seen in the VN assay but there was no observed differentiation between antigens in individual clades 5a.2, 5a.2a and 5a.2a.1.

### 2.4 Human Serology

Three serum panels taken from adult volunteers (18–64 years of age) over three consecutive vaccination campaigns were analysed: 2020 ahead of the NH 2020/21 season, 2021 for the NH 2021/22 season and 2022 for the NH 2022/23 season. Volunteers were immunised with inactivated vaccine antigens corresponding to egg‐propagated GM19 (clade 5a.1) in 2020, and cell culture–propagated Wsc19 (clade 5a.2) in 2021 and 2022. These antisera were used in HI assays with the homologous vaccine viruses and cell culture–propagated Wsc19 for the 2020 panel, and cell culture–propagated cultivars of Syd21 (subclade 5a.2a‐C.1) and Wsc22 (subclade 5a.2a.1‐C.1.1) for the 2021 and 2022 panels.

**FIGURE 2:**
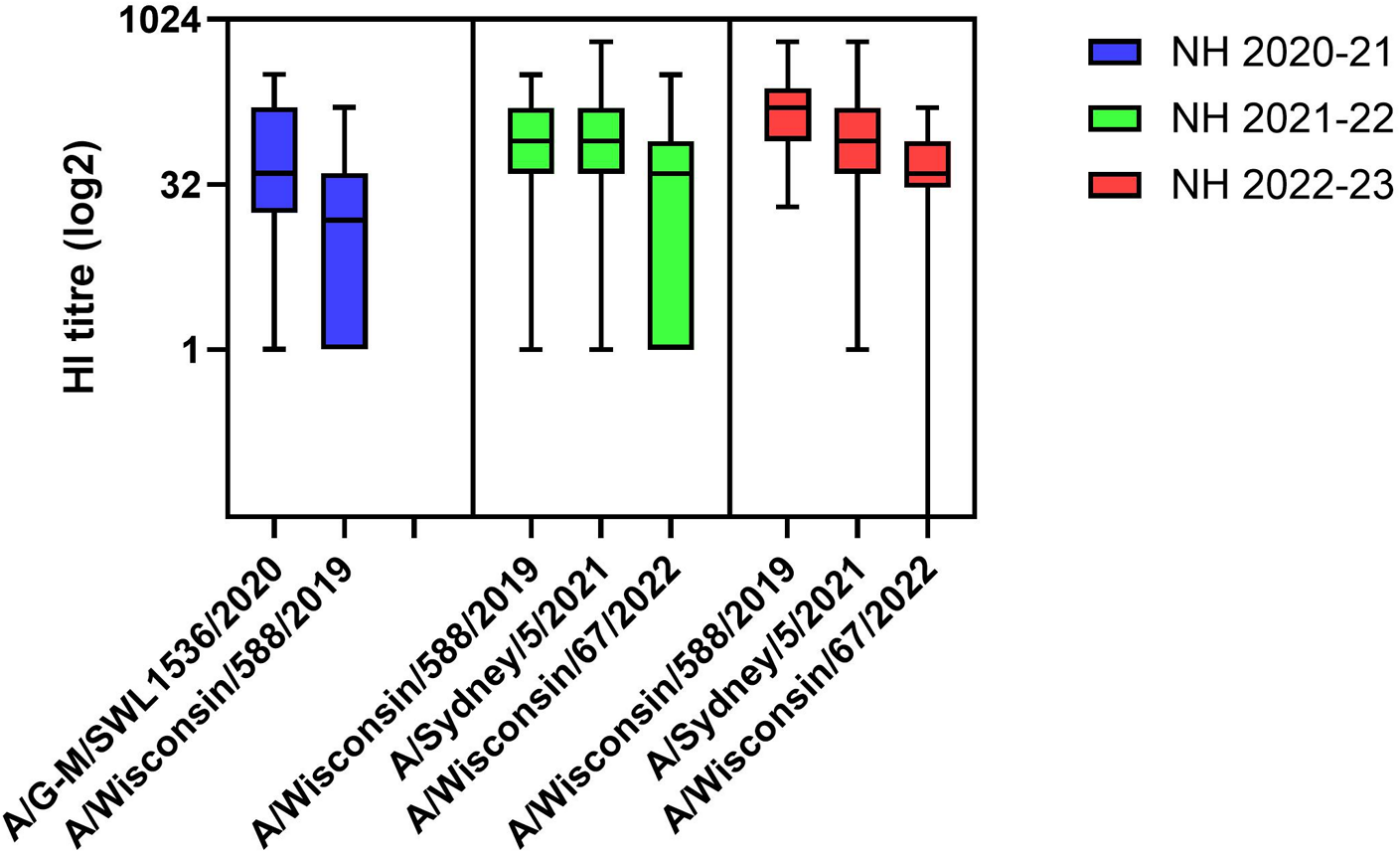
Postvaccination HI titres of three serum panels from healthy adults. Serum panels from three consecutive Northern Hemisphere (NH) vaccination campaigns: 2020 for 2020/21 season (in blue), 2021 for 2021/22 season (in green) and 2022 for 2022/23 season (in red). Each panel was tested against the A(H1N1pdm09) virus component of the vaccine for the season and test viruses as shown. Data are plotted on a log_2_ scale.

Nonparametric analysis of the paired postvaccination HI titres using a Wilcoxon matched‐pairs signed rank test showed that the human sera differentiated GM19 from Wsc19 (*p* < 0.0001) in the 2020 vaccinees. The sera from the 2021 vaccinees clearly differentiated Wsc22 from Wsc19 (*p* = 0.0005) but not Syd21 (*p* = 0.083), while the sera from the 2022 vaccinees differentiated both Syd21 (*p* = 0.0009) and Wsc22 (*p* = 0.0001) from Wsc19 (Figure 2).

Analysing individual serum responses showed that of the 22 volunteers vaccinated with egg‐propagated GM19 eight recognised Wsc19 at titres reduced fourfold or more. Of the 29 volunteers vaccinated with cell culture–propagated Wsc19 in 2021, 12 (41%) recognised Syd21 and 22 (76%) recognised Wsc22 at titres reduced fourfold or more, while of the 25 volunteers vaccinated in 2022, 9 (36%) recognised Syd21 and 13 (45%) recognised Wsc22 at titres reduced fourfold or more.

### 2.5 Comparative Analysis

The postinfection antisera from ferrets and pigs and the postvaccination human sera distinguished GM19 (clade 5a.1) from Wsc19 (clade 5a.2). The HA of this pair had the substitutions K130N, N156K, L161I, A187D, E189Q and V250A in HA1 and E179D in HA2 (Table 1), and four of these seven substitutions were mapped to antigenic sites: Sa (K130N and N156K) and Sb (A187D and E189Q). Five of these HA1 substitutions (not E189Q) were conserved in the remaining four members of the virus panel. The antisera from both ferrets and pigs did not differentiate Wsc19 from Syd21 (subclade 5a.2a‐C.1) in HI or VN assays, with Syd21 having HA1 substitutions K54Q, D94N, A186T, Q189E (reversion), T216A, E224A, R259K and K308R (Table 1). These antisera also did not distinguish Vic22 (subclade 5a.2a.1‐D), Lis23 (subclade 5a.2a‐C.1.9) and Wsc22 (subclade 5a.2a.1‐C.1.1) from Wsc19, despite viruses in these subclades differing in HA1 amino acid sequence at several sites including the substitutions T120A, P137S, K142R, K169Q, D260E and T277A (Table 1). Importantly antisera from both animal species did not clearly discriminate between Wsc19 and Wsc22 in contrast to the postvaccination cohorts of human sera in 2021 and 2022, which showed a reduction in human sera reacting to Wsc22 (Figure 2 and [2, 3]). This pair of viruses differed at nine HA1 residues K54Q, P137S, K142R, A186T, Q189E, E224A, R259K, D260E and T277A. The altered recognition by the postvaccination sera resulted in a recommendation to update the cell‐based and egg‐propagated vaccine strain components for the NH 2023/24 season [3].

These results indicate that, like postinfection ferret and swine antisera, postvaccination human sera (although with some degree of variation) are able to differentiate the clade 5a.1 vaccine virus from Wsc19 (clade 5a.2). Postvaccination serum panels as a whole, also differentiate the vaccine virus Wsc19 from Wisc22 (subclade 5a.2a.1‐C.1.1) and in one of the two seasons differentiated Wsc19 from Syd21 (subclade 5a.2a‐C.1) unlike the ferret and pig antisera. These results with human sera are consistent with published results from postvaccination human serum panels used in vaccine strain recommendations for the SH 2023 and the NH 2023/24 influenza seasons [2, 3]. Nevertheless, individual sera differed in their discrimination between viruses in the different subclades.

## 3 Discussion

Postinfection ferret antisera and postvaccination human sera are usually used to define the antigenic characteristics of human influenza viruses as they evolve. However, for some recent seasonal influenza viruses there has been a disparity in the recognition of viruses between human and ferret antisera, which has impacted on human vaccine strain selection, notably so for A(H1N1pdm09) viruses [2, 11, 12, 13]. Therefore, this study aimed to evaluate the immune response in pigs following infection with A(H1N1pdm09) human influenza viruses, to compare their recognition of recent A(H1N1pdm09) viruses with those of ferret and human antisera and to investigate whether postinfection pig antisera can enhance the antigenic analysis of human influenza A(H1N1pdm09) viruses.

The overall study findings revealed that pigs could readily be infected with seasonal human A(H1N1pdm09) influenza viruses. This was not unexpected and confirmed that pigs remain susceptible to contemporary human seasonal viruses descended from the 2009 swine origin pandemic virus and support ongoing detections of virus spillover from humans to pigs [14, 15]. Broadly, pig immune responses produced consistent antigenic profiles directly comparable to those of ferrets; although we obtained some altered characteristics when comparing neutralisation profiles for ferret and pig antisera between homologous and heterologous viruses, these were not correlated with subclade.

Although the pattern of recognition of ferret and pig antisera was similar, direct comparisons of the immune responses showed that while the pairs of pigs in this study had comparable homologous and heterologous HI titres, they were lower than those of ferret (approximately 16‐fold across all viruses, except for GM19, a virus antigenically very distinct from the other viruses examined), which reduces the dynamic range of the assays. When homologous titres in VN assays are compared, generally pig antisera titres were lower than those of ferret, showing pig‐specific reductions of 12‐ to 211‐fold for all but antisera raised against Syd21 (Table 2B). Such elevated HI titres in ferrets are a well‐documented feature of the model and reflect strong antibody responses following experimental infection of seronegative animals, resulting in highly focused primary antibody responses with very high titres [16]. In contrast, human serology studies report protective HI thresholds of approximately 1:40, with most postinfection or postvaccination titres for H1N1 strains falling below 1:320 and geometric mean titres often below 100 [17, 18]. These human ranges closely resemble those observed in influenza naive pigs experimentally infected with the human H1N1 viruses, where postinfection HI titres typically remain in the lower hundreds and rarely approach ferret values [19, 20, 21]. The exaggerated antibody magnitude in ferrets likely reflects high challenge doses, leading to strong systemic B cell activation and expansion, whereas pigs, as a natural host with immune responses and respiratory physiology more like humans, mount more moderate humoral responses. Therefore, while ferrets remain highly valuable for antigenic characterisation, the exact reasons for the absolute HI titres generated in this model are not fully understood and should be interpreted with caution when extrapolating to pigs or humans.

The utility of pig and ferret antisera in evaluating responses to phenotypic evolution of swine influenza viruses has recently been compared [22]. In that study, swine sera were produced following intranasal infection and subsequent intramuscular immunisation with the same virus dose formulated with adjuvant, whereas ferret sera were obtained after intranasal infection only. Despite these differences in immunisation/infection regimes, the breadth and relative magnitude of the antibody responses were equivalent in both hosts, as assessed by HI and VN.

We have previously demonstrated that monoclonal antibodies (mAbs) derived from A(H1N1pdm09)‐infected pigs recognised the same two major immunodominant HA sites, Sa (residues 160 and 163) and Ca (residue 130) [9]. In 2014, A(H1N1pdm09) viruses acquired antigenic drift through HA1 K163Q substitution which was not detected using ferret antisera [11, 23, 24, 25]. Whether mAbs generated from the infected pigs in this study would reveal the fine specificity of epitopes in Wsc19 and Wsc22 remains to be determined. By 2016, the dominant A(H1N1pdm09) viruses circulating had acquired an NQS glycosylation motif through HA1 S162N substitution, effectively masking the Sa antigenic site.

These results reaffirm the continued importance of the interface between humans and pigs for ongoing influenza virus evolution and potential antigenic divergence while retaining interspecies transmissibility. The genetic data obtained from viruses prior to infection and those shed 4 days after infection showed retention of consistent human‐derived virus genetic characteristics, supporting maintenance of similar viruses in pigs albeit without defining transmissibility between pigs. Given the high susceptibility of pigs to human A(H3N2) influenza viruses and the maintenance of such viruses many years after the antigenic variants have disappeared from humans [26], due to genetic drift, there is also merit in assessing comparative data between ferrets and pigs to discriminate antigenic drift leading to A(H3N2) vaccine strain changes.

This is the first study directly comparing ferrets and pigs as hosts to define antigenic reactivity and variation in human A(H1N1pdm09) influenza viruses. We were able to show little variation qualitatively between the two animal hosts when using postinfection antisera to discriminate between viruses. Therefore, pigs could offer a qualified alternative for measuring antigenic variation in human influenza A viruses. This study also broadens the scope of pigs to serve as an animal model for human influenza for evaluating immune responses and vaccine studies as one of the milestones for improved influenza vaccine research [10].

## 4 Materials and Methods

### 4.1 Virus Propagation

Viruses provided by the Francis Crick Institute (Table 1) were propagated in Madin–Darby canine kidney (MDCK) cells. MDCK cells were cultured in T75 flasks with Dulbecco's modified Eagle's medium (DMEM; Sigma Aldrich), 10% fetal bovine serum (FBS; Gibco) and 1% penicillin and streptomycin (PS, Sigma Aldrich). Cells were inoculated with 2 mL of a seed virus diluted in DMEM + PS and incubated for 30 min at room temperature before adding 10 mL of DMEM + PS + 2 μg/mL (TPCK)–treated trypsin and incubated for 2 to 3 days at 37°C with 5% CO_2_. The gene sequence of each virus stock was confirmed before use.

### 4.2 Influenza Infection of Pigs

Twelve 6‐ to 8‐week‐old, outbred Landrace × Large White pigs (6 female and 6 male) were obtained from a commercial high‐health status herd. The pigs were screened by ELISA for the absence of serum IgG titres against A(H1N1pdm09) and A(H3N2) virus. For each study virus two pigs, with one male and one female randomly divided per group, were infected. After a week of acclimatisation, the two pigs in each study group were inoculated intranasally using a mucosal atomisation device (MAD, Medtree, UK) with 2 mL (1 mL per nostril) with either A/Guangdong Maonan/SWL1536/2019 (GM19; 1.8 × 10^5^ PFU/pig) or A/Lisboa/188/2023 (Lis23; 5 × 10^6^ PFU/pig) or A/Sydney/5/2021 (Syd21; 8 × 10^6^ PFU/pig) or A/Victoria/4897/2022 (Vic22; 4 × 10^6^ PFU/pig) or A/Wisconsin/588/2019 (Wsc19; 1 × 10^6^ PFU/pig) or A/Wisconsin/67/2022 (Wsc22; 1.5 × 10^6^ PFU/pig) (Figure 1A). At 14 DPI, pigs were humanely euthanised with an overdose of pentobarbital sodium anaesthetic and clotted blood and bronchoalveolar lavage fluid (BAL) were collected to assess immune responses to the viruses.

### 4.3 Sampling of Study Pigs

NSs (two per nostril) were taken from pigs 4 days before infection, and 2, 4 and 7 DPI to assess virus shedding. The swabs were placed into 2 mL of virus transport medium comprising tissue culture medium 199 (Sigma‐Aldrich) supplemented with 25‐mM 4‐(2‐hydroxyethyl)‐1‐piperazineethanesulfonic acid (HEPES), 0.035% sodium bicarbonate, 0.5% bovine serum albumin, penicillin 100 IU/mL, streptomycin 100 μg/mL, and nystatin 0.25 μg/mL, vortexed and centrifuged to remove debris, then stored at −80°C for subsequent virus titration. Blood samples were collected in BD Vacutainer SST serum separation tubes at the start of the study (prior to challenge) and at 14 DPI. BAL was collected from the entire left lung with 0.1% BSA in PBS. BAL samples were centrifuged at 500 × g for 5 min, and the supernatant was aliquoted and frozen for further analysis.

### 4.4 Plaque Assay

Virus titres in NSs were determined by plaque assay on MDCK cells as described previously [27]. Plaques were visualised by staining the monolayer with 0.1% (v/v) crystal violet containing 20% methanol (Merck) and expressed as plaque forming units (PFU) per ml of NS eluate.

### 4.5 Virus Whole‐Genome Sequencing

Viral RNA extraction and influenza virus whole genome sequencing were performed as previously described [28]. Raw fastq sequences were then imported into Geneious Prime software and trimmed to remove primers and aligned to whole genome reference sequences (GM19 [EPI_ISL_377080], Syd21 [EPI_ISL_19193801], Wsc19 [EPI_ISL_404527], Lis23 [EPI_ISL_18788764], Wsc22 [EPI_ISL_15928538], Vic22 [EPI_ISL_19192400]).

### 4.6 Ferret Antisera Production

Postinfection ferret antisera were generated by intranasally inoculating ferrets with 500 μL (250 μL per nostril) of virus. Two weeks after inoculation, sera were collected.

### 4.7 Human Serology Panels

Pre‐ and 2‐week postvaccination human sera were collected over three consecutive years from healthy volunteers working at the Francis Crick Institute. Inclusion criteria included the receipt of a seasonal vaccine, being employed at the Francis Crick Institute and consenting to give a prevaccination and postvaccination blood sample. No details of previous vaccination/infection history were obtained. Samples were collected in BD Vacutainer SSTII tubes and centrifuged for 10 min at 2000 rpm to separate red and white blood cells from the serum. The serum supernatants were transferred into separate tubes and heat inactivated at 56°C for 30 min before storage at −20°C.

### 4.8 HI Assay

Ferret and pig antisera were heat inactivated at 56°C for 30 min and treated with receptor destroying enzyme (RDE) (Denka Co.). RDE‐treated sera were diluted to a working dilution of 1:20 in PBS with 0.1% azide. Haemagglutination and haemagglutinin inhibition (HI) assays were performed according to standard methods using suspensions of turkey RBCs (0.75% v/v) [29]. Four haemagglutination units were used in all HI assays.

### 4.9 VN

Viruses were titrated to determine the dilution at which 50% of cells within a well of a 96‐well plate were infected; these dilutions were used in the VN assay as previously described [30].

## Author Contributions

**Ruth Harvey:** writing – original draft, writing – review and editing, funding acquisition, methodology, formal analysis, data curation, investigation, supervision, software. **Tiphaine Cayol:** investigation, methodology, formal analysis, writing – original draft, data curation. **Basudev Paudyal:** investigation, validation, methodology, formal analysis, supervision, writing – original draft, data curation. **Alice Lilley:** data curation, formal analysis, visualization, validation, methodology. **Christine Carr:** data curation, formal analysis, visualization, methodology. **Catherine Hatton:** methodology, visualization, data curation, formal analysis. **Emily Briggs:** methodology. **Rodney S. Daniels:** conceptualization, methodology, investigation, funding acquisition, writing – original draft, writing – review and editing, project administration, supervision. **Samuel Richardson:** data curation, investigation, methodology. **Thomas P. Peacock:** methodology, investigation, data curation, formal analysis, supervision, writing – original draft, writing – review and editing, software. **Nicola Lewis:** conceptualization, methodology, funding acquisition, writing – original draft, writing – review and editing, project administration, supervision, software. **Ian Brown:** conceptualization, investigation, funding acquisition, writing – original draft, writing – review and editing, methodology, supervision, formal analysis, software. **John W. McCauley:** conceptualization, investigation, funding acquisition, writing – original draft, writing – review and editing, project administration, supervision, software. **Elma Tchilian:** conceptualization, investigation, funding acquisition, writing – original draft, writing – review and editing, project administration, methodology, supervision, validation, visualization, resources.

## Funding

This work was supported by Biotechnology and Biological Sciences Research Council (BB/X511134/1, BBS/E/PI/230002A, BBS/E/PI/230002B, BBS/E/PI/23NB0004, BBS/E/PI/23NB0003, BB/Y007298/1), Francis Crick Institute (CC1114), Medical Research Council (MR/Y03368X/1, CC1114), Bill and Melinda Gates Foundation (OPP1215550) and Wellcome Trust (CC1114).

## Ethics Statement

The pig studies were approved by the ethical review processes at APHA and the Pirbright Institute in accordance with the UK Government Animal (.Scientific Procedures) Act 1986 under Project License PP2064443. Collection of human prevaccination and postvaccination serum samples was approved by The Francis Crick Institute Internal Ethics Review Committee, registration number: 2019 FC5.

## Conflicts of Interest

The authors declare no conflicts of interest.

## Data Availability

Data generated or analysed during this study are available within the article. Additional supporting data are available from the corresponding authors without undue reservation.
